# Research on the Integration of Media Literacy Innovative Concept and Entrepreneurship Education and Digital Dynamic Creative Expression Talents

**DOI:** 10.3389/fpsyg.2021.728182

**Published:** 2021-10-18

**Authors:** Jie Zhang, Mingming Zhang, Yaqian Liu, Ruimin Lyu, Rongrong Cui

**Affiliations:** ^1^Department of Digital Media Arts, School of Design, Jiangnan University, Wuxi, China; ^2^Graduate School of Management, Management and Science University, Shah Alam, Malaysia

**Keywords:** media literacy, innovative concept, entrepreneurship education, dynamic design talent, innovative expression

## Abstract

The rapid development of digital technology has created a variety of forms of digital media. In these emerging media, with the support of high-performance computers, increasingly dynamic performance has become possible, and the public has cultivated a preference for dynamic content cognition. This study, based on the basic characteristics of visual perception to the cognition of motion form, aims to cultivate the cognitive literacy of pan-digital media with innovative concepts and entrepreneurship education and to explore the cognition and innovative expression methods of dynamic language in digital design. The research leads the static oriented morphological exploration and expression to the dynamic expression and thinking of the same concept object. The basic thinking steps for students from “static” to “dynamic” are established, and students are encouraged to use “Synesthesia,” “metaphor” and other methods to carry out a “dynamic expression” level of emotional association. In the experiment, two different ways of design expression, static and dynamic, are required to design and evolve graphics. In this study, 50 freshmen were selected as the training objects for the planning and training of design thinking and performance means. In the visual elaboration and expression of the inner emotion of the same content with innovative concept and entrepreneurship education, not only should the changes and combinations of the graphics be innovated, but the emotional characteristics of the more abstract graphics should be explored as well. The feedback data of students’ thinking and cognition differences in the two stages of expression were obtained through a questionnaire and analyzed and compared. The experimental results show that after the training, students’ ability to develop innovative concepts and entrepreneurship education through dynamic expression, consciousness and perception were significantly improved. This research also provides a new vision and specific implementation method for the future training of digital dynamic innovation expression ability and the cultivation of innovative concepts of digital media literacy and entrepreneurship education.

## Introduction

### Motion Practice and Holistic Educational Thinking in the Basic Education System of Bauhaus

At the Bauhaus Week and Bauhaus exhibition in 1923, “Art and technology-a new unity” was declared a slogan, and “The combination of art and technology (machine)” became the most important ideology. The rise of constructivism (art) had a great influence on Bauhaus design, and constructivism (art) emphasized movements in space and broke the original single static way of expression (Birringer, 2013). Under the influence of both art and technology, later in a series of educational practices, painting, sound, music and other data are subsequently imported into a computer to explore the whole expression formed by multimedia and multichannels to construct a comprehensive synthetic art work (Chen and He, 2013). The concept of motion was originally carried out in Bauhaus’s paintings; through the use of the dynamic composition and dynamic perspective of visual features in painting, the static image becomes more vivid and energetic. As a “moving image” with an element of time, László Moholy Nagy’s work is outstanding. We can find his thoughts and practice in “Malerei, Photographie, Film (1925)” and “Vision in Motion” (1947). His experiments in synthetic art and motion led to a new kind of artistic literacy (Foster et al., 2006). Due to the rise of multimedia today, Moholy Nagy has introduced new creative techniques in the fields of art and education (Nelson, 2006). In addition, holistic educational thinking in Bauhaus basic design education also emphasizes the integration of “body and mind” into innovative basic art education, which compensates for the shortcoming “lack of practical experience” in works produced by digital technology without using physical materials in the aspect of multimedia expression (Gropius, 1992). Guided by this kind of thinking, multimedia artwork requires the creator to have a rich experience in generating ideas, planning, finding materials, assembling and editing his target image.

### Characteristics of Contemporary Digital Media and the Basic Education of Art Design

The French scholar Marco Diani once stated “In postmodern society, one of the main characteristics of the dominant structure that dominates people’s work is the ubiquity and adaptability of new technologies. From the development of human work to machine work, and then developed to computer work, during which technological changes have taken place rapidly, which led to the changes of individuals and groups to adapt to their special working environment. Design has become a more complex and multidisciplinary activity than it was not long ago” (Wang, 2016). Since the twenty-first century, China has new requirements for the development of ideology on a certain economic basis. In addition, innovative entrepreneurship education in universities is an educational model that is compatible with socioeconomic development and education reform, which should reflect the integration, innovation, and application of knowledge (Dieser and Christenson, 2017). The professional and basic ability of the emerging digital media art design is the most intuitive in assessing the impact of the changes in new media and new technologies. In the traditional sense, plane composition, three-dimensional composition and color composition are static forms in a specific space. In digital media design, there are few static forms, and any form of composition will add a feature of the time dimension. The appearance of this characteristic directly causes continuous change in the interrelationship among single elements in a specific space. If the form change in a plane space or three-dimensional space is regarded as a frame on the time axis, then the expression of a period of movement is a space-time matrix composed of a plane or three-dimensional space. In this matrix, a single element that used to only have a relationship with adjacent elements in space will now have a relationship with “itself” before and after this moment. The visual psychological impression generated by this change is obviously more intuitive and stronger than that expressed in the form of static space (Hayles, 2012).

Image as a means of communication and vision as a means of understanding the world are particularly prominent in the lives of contemporary young people. Today’s students grow up in an environment saturated with images, and their communication and practice often use vision as a medium (Brumberger, 2019). Artistic media literacy includes an understanding of the characteristics of the medium and comprehensive ability to effectively create and express intentions using image media (Motomura, 2003). In the image age, people’s visual literacy reflects the possibility of receiving and cognition of external information. Media art, including product design, packaging, painting, sculpture creation, architecture and landscape design, require visual imagination and thinking. The expression of motion seems to be promoted by the development of the media that is spawned by the development of information technology, but from the perspective of people relying on vision to obtain information, it coincides with an improvement in people’s visual literacy. Artistic media literacy includes the understanding of media characteristics and the comprehensive ability to use image media to effectively create and express intentions. Visual literacy is directly related to the behavior of reading images. When it was first defined, it crossed disciplines and backgrounds in the practice of higher education (Kędra, 2018). When people see a certain thing, they form a “posture” by the visual perception of the thing, and this “pose” and “build momentum” are actually determined by people’s visual imagination and thinking. Graphic images under media art have become the most important expression tool for interpreting and disseminating culture, and text has become the annotation and supporting role of image symbols. Visual temptation and interpretation have never been more important. In the context of contemporary digital media visual communication, the training of digital media art design must be geared to the increasing visual literacy of the masses and meet the expression needs of emerging media (Messaris, 2012). In this way, the visual expression of digital media can be comprehensively considered and interpreted from a dynamic perspective and integrated into the fundamental teaching of basic elements and design forms. Visual literacy skills are learnable, teachable, and can be developed and improved, conscious visual literacy training can help improve learners’ visual literacy. Through the training of coping with characteristics, students will open the door to digital media art design expression and cultivate them to adapt to a new visual habit. In the current information age, the education forms of Chinese universities have also changed, and research on the cultivation of digital dynamic creative expression literacy with the concept of innovation and entrepreneurship education is actively attempted under this background (Sun, 2020).

## Materials and Methods

The purpose of this research is to discuss the cultivation of talents for digital dynamic creative expression in the context of the practice of visual literacy education, especially for students majoring in digital media art, and to provide relevant professional educators with methods and reference significance in future curriculum design. For this reason, in the early stage, I designed the overall framework of the course based on the characteristics of digital media art professionals and the new media needs, and transformed the dynamic needs of students’ visual literacy into basic course training, and accompanied the course. During the process, 50 freshmen were used as the training objects, 30 of them participated in every link of the course training, and they were trained by means of content selection and form application; the other 20 were the control group, they only received the regular plane composition, color composition and other composition training, did not receive relevant dynamic expression exercises, and at the end of the course, through questionnaire surveys and interviews, the different impressions of the two groups of students on dynamic expression were explored and compared.

The basis for the overall framework design of the course stage and the evaluation results of students’ dynamic expression effect are as follows.

### To Develop the Basic Design Capabilities of Digital Media for New Media Needs and Characteristics

Digital Media Art was established with the emergence of new media and the formation of digital industries. Its main purpose is to integrate design thinking and artistic expression into new digital media Similar to the Bauhaus basic education system, which revolves around the urgent needs of machine production after the second industrial revolution. The fourth industrial revolution is also the formation of the information age, which has given birth to a series of digital media surrounding social and technological changes, such as digital communication, digital expression, and digital art. In such a medium, design is an innovative driving force; it can change and optimize a series of systemic relationships in the physical world and establish and optimize system relationships for digital products and digital artistic expressions in the digital world. With the development of the information age, the digital world will become a mirror image of the real world and has more possibilities than the real world. Therefore, design as a systematic way of thinking will inevitably become an important attitude in the development of the digital world (Sevaldson, 2014).

**FIGURE 1:**
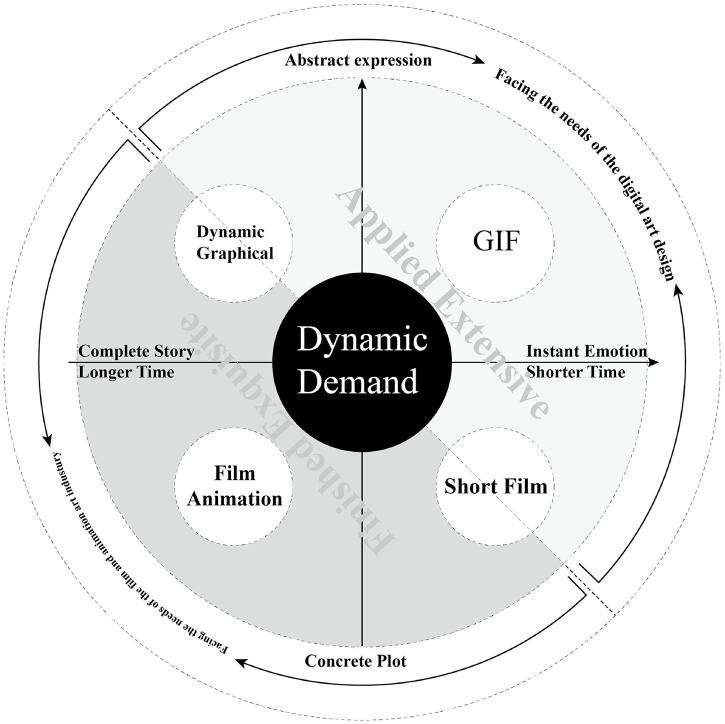
Dynamic form for dynamic demand.

In the gradually forming digital world, new media are constantly appearing. The previous computer was the main interface from the real world to the digital world. With the development of software and hardware, the form of computers has gradually presented the trend of miniaturization, decentralization and implantation. The number of digital media is far greater than that of traditional media, and the form of digital media is far greater than that of traditional media. Due to the enhancement of computability, the expressible dimensions of digital media are broader than those of traditional media (Osadcha and Baluta, 2021). Among them, the most important feature is that the communication channel of traditional media is single, while the communication channel of digital media is multidimensional. The expression of traditional media is static, and the expression of digital media has a time dimension, that is, it is dynamic. Focusing on this main difference, in contrast to the current basic courses of Digital Media Art Design in China, plane composition, three-dimensional constitution and color composition are employed as training of modeling, which to some extent disregards the important characteristics of contemporary digital media. Bauhaus’ basic education system of design introduced the study of “dynamics” earlier through the study of vision. In the process of the artist’s research and practice of motion vision, even in traditional medium, “vision in motion” was also shown (Itten, 1975). In the basic design courses for the new digital media, the needs and characteristics of the medium should be taken as a new aspect of design basic education, and more exploration and practice should be carried out to cultivate students’ cognitive literacy of pan-digital media with innovative ideas and entrepreneurial education, as shown in Figure 1.

### Transform the Dynamic Demand Into the Concrete Basic Course Training

#### Selection of Content

**FIGURE 2:**
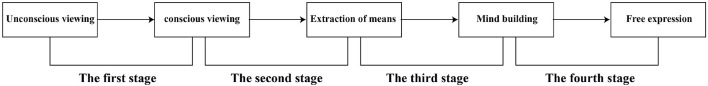
Four basic stages of training.

The training of dynamic innovative expression is set in the latter stage of modeling training, and the cognition and training of static modeling in the previous stage of the course are utilized as a graphic basis of dynamic expression. To achieve the most fundamental training of dynamics, dynamic exercises are combined with abstract graphics, and emotion is expressed through the unified arrangement of and change in abstract elements of points, lines and planes. The training of the course is based on four basic stages, as shown in Figure 2: The purpose of the first stage of training is to transform unconscious viewing into conscious viewing; the purpose of the second stage is to extract means based on conscious viewing; in the third stage, the establishment of dynamic expression thinking is carried out after having certain basic expression means; the final stage tries to integrate different emotions and feelings into the depiction of time, and uses the movement of abstract objects to express certain emotions.

**FIGURE 3:**
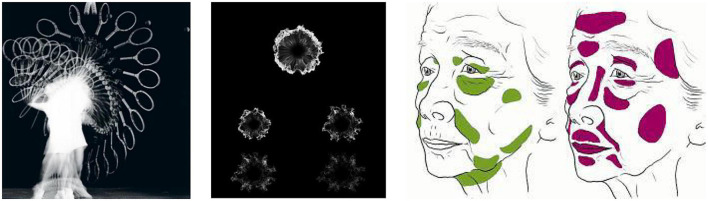
Observation and reflection on morphological changes in time dimension.

The first stage: Reshape the observation method. In this stage, breakthrough training is carried out for the inertial visual observation method. Through the observation of natural objects from different angles, growth stages, and decomposition methods, new observation perspectives are cultivated, especially the perceptual cognition and description of the influence of time on form. Establish an internal perspective of “change” in the process of static depiction, as shown in Figure 3.

**FIGURE 4:**
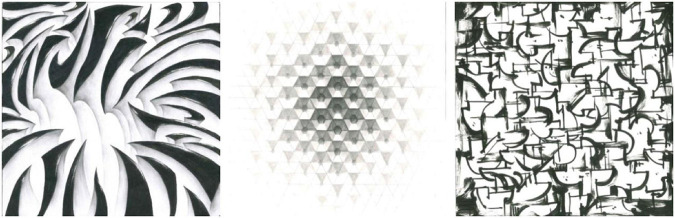
Abstract extraction of physical form.

The second stage: Through the training of graphic abstraction and simplification, understand the rules of formal beauty, learn the abstract extraction of concrete forms, achieve the ability to abstract the characteristics of concrete objects into a composition picture with certain emotional characteristics, and shape the total expression of the picture by using the emotional association and metaphor characterized by points, lines and planes, as shown in Figure 4.

The third stage: establish the thinking of dynamic expression. The motion of form is the sequential display of different form states on the time axis, and the position, mutual relations and changes of form are organized to constitute a display for a period of time. The course chooses the graphics composed of bones as the basis, simplifies the complex expression of movement by means of different analysis methods of bones, regulation of the change order of unit shape, setting of the initial state of movement, etc., and strengthens the application of number and order logic in expression by searching for the nature of dynamic change: time, position, and order.

The fourth stage: Based on mastering certain thinking methods, rhythm, direction, color, acceleration and deceleration and other motion elements are systematically combined to express a certain emotion.

#### Selection of Forms

Dynamic design is a broad concept, especially the mature of information technology and media technology, which makes people more inclined to obtain information from the media in a dynamic way. However, due to the ambiguity of the boundary in the area from animation to dynamic design, in the field of art design, dynamic design relies on the concept of pananimation expanded by the basic teaching of animation (Song, 2021). However, as dynamic application subdivisions become increasingly mature, dynamic design needs to address the different characteristics and specific purposes of diverse media. Considering the curriculum setting; combining the characteristics of the integration of art and engineering and the needs of diverse media expressions, guidance for entrepreneurship education and innovative concepts; and focusing on the dynamic expression of arrays, through the connection with the grid composition part of the sculpt foundation, the composition logic and the motion organization logic in the motion design are consciously implanted into the dynamic expression of graphics. The implementation of related courses is also carried out by selecting the following specific forms.

**FIGURE 5:**
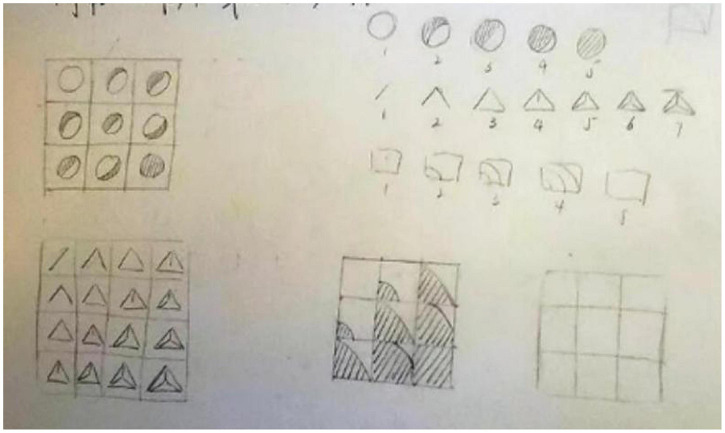
Methods of practicing orderly organizational dynamics with simple graphics.

(1) Analyze the composition logic of existing art works: First, observe the changes in the static grid composition and establish the rules; second, explore multiple observation angles of the static composition; and last, use numbers to mark the changes.
(2) Digital logic in team motion practice: Through experiential exercises to strengthen the relationship of motion changes between individual elements and the group. The students are divided into groups of 7 people, and each student represents a unit element. The group will discuss for approximately 10 min to determine the motion form of the unit and the sequential rule of group motion, form an overall scheme and perform live after discussion and confirmation.
(3) Organize motion with digital logic in graphic practice: After team practice, based on an understanding of the sequential rule of group motion with digital markers, the corresponding relationship between numbers and graphics is set, and the digital composition logic in the existing art works is applied to organize the total motion of the grid pattern, as shown in Figure 5.

(4) Software practice of organizing motion with digital logic: the self-made program by unity3D is utilized to carry out the tentative design of motion. In the homemade program, structured settings according to the grid form and digital logic allow students to design the sequential rule of grid motion by setting parameters. Based on the first two steps, the circular motion of the unit shape and the numerical law of the grid are applied to set the total motion style. Convolutional neural networks are utilized to learn and analyze the emotional characteristics of literati paintings.

#### Design of Training Focus

**FIGURE 6:**
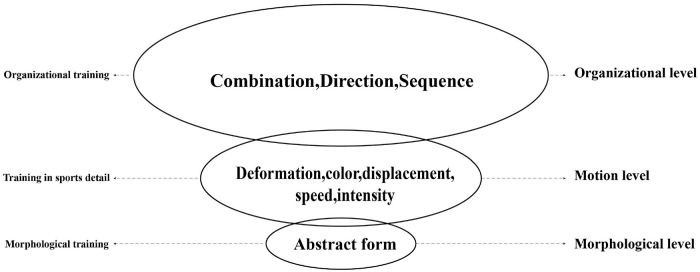
Hierarchy of training priorities.

The focus is to cultivate the ability to express emotional characteristics using the motion state of abstract morphological elements in successive training and the new visual thinking habits that are adapted to dynamic expression formed in the process. (1) Pay attention to the changes in the morphological elements on the time axis; (2) Focus on the group performance of a single cyclic dynamic; and (3) Abstract mimicry in motion, as shown in Figure 6.

#### Use of Auxiliary Tools

**FIGURE 7:**
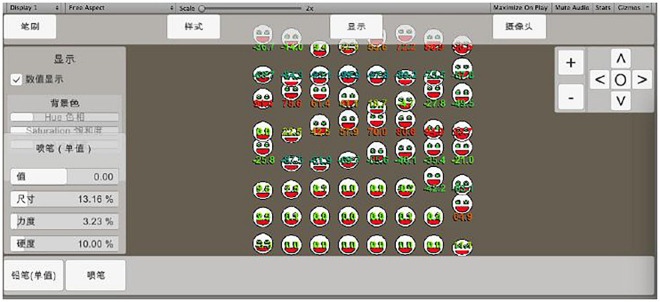
A method for dynamic expression of grid composition in homebrew.

A self-made program is selected as an auxiliary tool for training. The tool has a basic grid setting function, which simplifies the complex array movement into the adjustment of “size,” “strength,” and “hardness” in units of “elements” to form a basis for the dynamic expression diversity of grid composition, as shown in Figure 7.

#### Semistructured Interview

The course uses sample questionnaires and sample interviews to conduct surveys. The main purpose is to explore the status of students after learning: (1) cognition and awareness of motion expression and (2) the connection between motion expression and emotional communication. Thirty of the students who participated in the survey participated in every practice part of the course. In addition, a control group was also set up, with the remaining 20 people in the control group, and the main contents of routine composition training were continued for them. The questionnaire and interview process mainly explored students’ sensitivity to the dynamic characteristics of media and cognition of emotional expression from two aspects—observation of motion and dynamic expression of emotion—to understand students’ impression of dynamic expression with the innovative concept and entrepreneurship education.

## Results

### Output of Innovation Results

**FIGURE 8:**
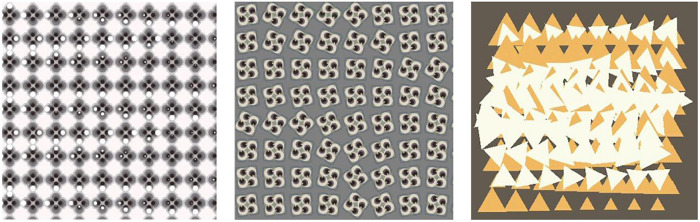
Work 1–3.

**FIGURE 9:**
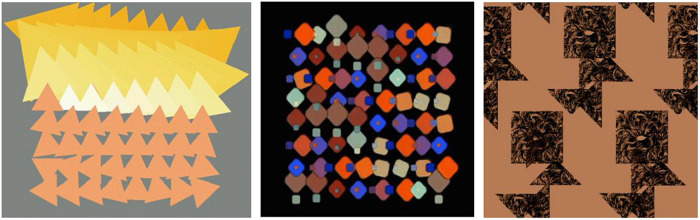
Work 4–6.

Through the continuous training of the whole idea, a number of exploratory works were produced. According to the unified requirements, the students carried out the design of the abstract graphics from the morphological level to the organizational level and tried to express a certain degree of emotional characteristics through the state of motion. For the final work, most of the students choose computer programs such as processing program or unity3D to realize their creative expression. These programs can realize the dynamic expression of graphics faster by writing code, and are unanimously welcomed by students. During this process, the students have to consider the richness and beauty of the motion in the array and how to form the emotional characteristics of the target through organization of the object’s motion order. Work 1–2 is a relatively typical array composition that uses the most basic equally spaced grid, and the dynamics of the graphics subject are mainly embodied inside the unit shape. Compared with work 1, work 2 rotates the unit shape more in the direction, which enables the dynamics of Work 2 to have richer visual changes with a stronger dynamic. Because of the difference in sequence, the motion process becomes more integrated. Work 3–4 is an attempt at different dynamics of the same set of basic forms. Compared with Work 3, Work 4 emphasizes the sense of order of deformation and color changes. However, due to the relatively single combination of unit shapes, although the visual impact is stronger, the dynamic level is relatively lacking, and the imbalance and instability of the graphics are formed in the middle. Work 3 has a reasonable sense of visual balance and richness, but due to the conservative dynamic design, the emotions conveyed dynamically are also conservative. Work 5 conveys the dynamic feelings of lively and nifty, a combination of large and small squares, and a jumping color relationship. Both graphics and color create a sense of dynamism, and their synchronization weakens the rigidity produced by the alignment. Work 6 is constructed from the shape formed after the picture is cut, and the main motion is the relative linear motion of the left and right. During motion, the triangles on the two sides will form a new graphic relationship when they meet, forming an interesting and dynamic visual form and greatly weakening the sense of existence of the grid due to the large range of dynamics, as shown in Figures 8, 9.

**FIGURE 10:**
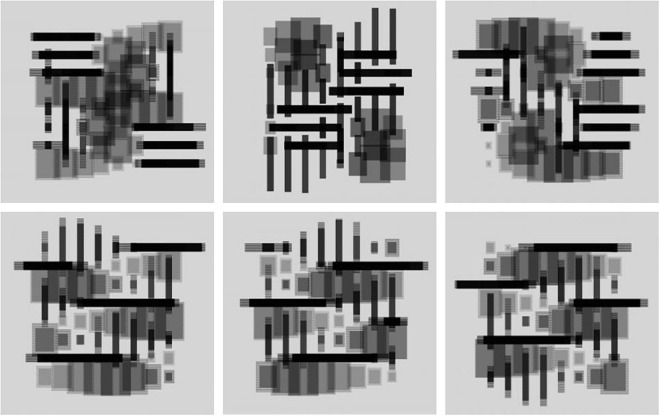
Work 7.

**FIGURE 11:**
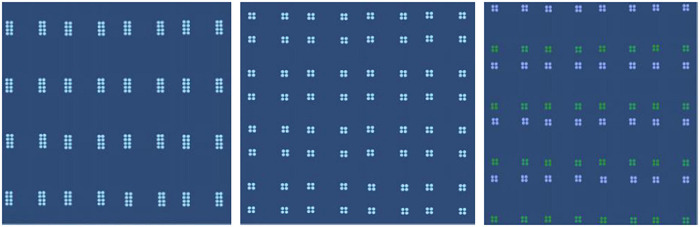
Work 8.

Work 7 is a set of dynamic grid graphics composed of lines and surfaces with transparency. The semitransparent graphics are stacked in the dynamic process to form a certain spatial front-to-back relationship, and the interspersion of lines and surfaces enriches the interrelationship in the dynamic realization process. Through the setting of different rhythms, an emotional rhythm is produced, and the rich changes also weaken the rigid mood created by the sense of grid. “Sense of space,” “uncertainty of form,” “emotional rhythm,” and “relative balance in dynamic” all render this group of works impressive, as shown in Figures 10, 11.

Compared with work 7, work 8 respects the grid form, using the simplest point arrangement as the basic graphics. However, in the process of dynamic expression and through the horizontal and vertical spacing changes and color changes, the neat emotions are expressed and some unexpected changes are also produced. The convergence and separation of points comprise the main content of morphological changes. However, the figure-ground relation formed by different combinations can also create graphical interest in the dynamics, thus forming certain emotional characteristics.

### Embodiment of Consciousness and Ability

After the course, set corresponding questions for students’ awareness, preference and sensitivity to motion observation and related aspects of dynamic expression of emotion, and make statistical charts based on relevant data. The questionnaire statistics for the course reflect, to a certain extent, the accumulation of students’ awareness and abilities in all aspects after the course.

First, it is the sensitivity study of dynamic feature observation, in the figure, the solid line is the experimental group, and the dotted line is the control group. First, it can be seen that the vast majority of people in the experimental group have a certain awareness of motion observation, but less than 10% have a stronger awareness. In the control group, the overall awareness of observation was not much different from that of the experimental group. However, there were two extreme groups: the first extreme group was characterized as completely “not feeling,” and the second extreme group was characterized as not “full of feeling.” People in the neutral zone were slightly more than 10 percent less likely than those in the experimental group to have awareness of motion observation. This set of data reflects that in the contemporary digital media environment, regardless of whether they participate in the course, the general population has a certain awareness of dynamic observation or a certain experience of observation.

**FIGURE 12:**
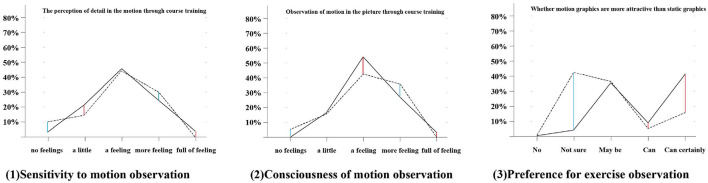
Line graphs of the two groups’ preferences for dynamic observation.

Figure 12 (2) shows the preference of the two groups for dynamic observation. The experimental group showed a distinct and positive preference for the attraction of dynamic observation. More than half of the control group showed a wavering preference between dynamic observation and static observation. Students’ ability of dynamic expression of innovative ideas and entrepreneurship education has been significantly improved. Figure 12 (3) mainly presents two groups of sensitivity characteristics for dynamic observation. Similar to the first figure, this figure presents a “head-to-tail” contrast. The trend of the curves of the two groups exhibit minimal difference. However, 10% of the control group and less than 5% of the experimental group chose the “not feeling” option, whereas approximately 5% of the control group and none of the experimental group chose the “full of feeling” category. In combination with the first figure, it can be inferred that the centralized dynamic training in the course can improve the attention of the subjects to the details in motion to a certain extent. However, the formation of literacy is affected by many existing digital media expressions, and the attention to dynamics changes with the changes in the surrounding information environment.

**FIGURE 13:**
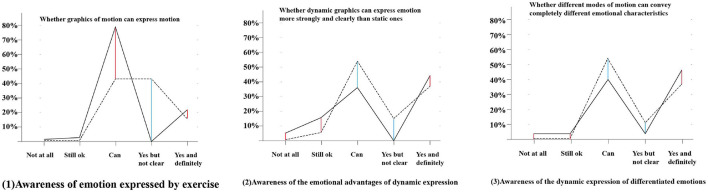
Line graphs of the differences in the perception of dynamic expression of emotions between the two groups.

Second, in digital media art works, a large part of the artistry of dynamic expression lies in the interpretation and expression of emotions. The experimental group in Figure 13 (1) showed a clearer understanding of the dynamic expression of emotions. The control group had a certain cognition but showed a greater sense of uncertainty, with 80% of the people expressing clear possibilities accounting for approximately 20% of the total population. Figure 13 (2) shows feedback on the advantages of dynamic expression in conveying emotions. The experimental group as a whole showed a strong sense of clarity. The control group also shows a certain degree of uncertainty, but the total number of people in the positive cognition exceeds the experimental group, and some people in the experimental group do not think that dynamic expression has advantages. Figure 13 (3) shows an understanding of the dynamic expression of differentiated emotions. The appearance presented in this set of data is similar to Figure 2. Similarly, most people in the experimental group showed a relatively firm sense of clarity, while the positive cognition of the control group was higher than that of the experimental group. As seen from the data in Figures 2, 3, the training of the course did not greatly promote the basic understanding of the “dynamic transmission of emotion” but strengthened the certainty of the dynamic expression of emotion and its advantages in the fuzzy media experience. Therefore, it can be seen that the choice of the experimental group is more affirmative, while the control group’s options are more ambiguous.

## Discussion

### Analysis of the Main Causes of Group Differences

The experimental group and control group in this practice mainly reflect the two aspects of group differences. The experimental group gave moderate recognition of “self-dynamic expression cognition,” and the peaks mainly appear around the more moderate options, while the control group gave more extreme recognition to this, and more “extreme negative” and “extreme positive” appear. On the other hand, the control group was approaching the experimental group in terms of the consciousness of “expressing emotions dynamically,” that is, the exploration and attempt in the course did not make the students in the experimental group have a clearer and stronger consciousness of the “dynamic expression of emotions.” In contrast, although the control group has not been exposed to the corresponding exploration and attempt, a considerable number of people have a relatively clear sense of identity for the dynamic expression of emotions. The differences between these two aspects may be attributed to the following reasons:

(1) Based on the original modeling training part, the dynamic expression of static graphics was extended in the course. Before exploring the dynamics, the experimental group went engaged in the “abstract study of form,” the “study of form change in the time dimension,” the “research of dynamic methods and steps,” “study on dynamic decomposition of self-substitution” and a series of process. The links of dynamic expression from observation, decomposition, and experience to hands-on reality further refine their cognition of the ways and steps of dynamic expression. Therefore, the cognition and denotation of boundaries may help form a relatively cautious attitude to “whether they think so.” In the control group, the cognition of dynamics is completely derived from life experience, and the lack of cognition of details may increase the “black or white” in the grasp of the general direction, forming a certain polarization trend.
(2) The second difference between the two groups was the similarity of identification consciousness between dynamic expression and emotional expression. In the original hypothesis, the experimental group and control group may have great differences in the awareness of “dynamic expression” and “dynamic expression of emotions.” The result was the opposite: the two groups did not widen the gap in proportions, and the control group appeared to be “more confident” in some ways than the experimental group. This difference is most likely due to today’s digital environment and the ubiquitous “multimedia” environment. Although the control group was not guided by the course, dynamic content had an imperceptible influence when contacting various digital media in daily life. If the “era of reading pictures” was formed with the prevalence of graphic language, then the popularization of digital media has brought us into the “dynamic era.” The perception and impression of the dynamic have become the “self-quality” of the group that has grown up in a digital media environment (Pulley, 2020). This accomplishment will further promote the development of “dynamic innovative expression” in a larger range and higher artistic level.

### Limitation of Selecting the Training Item of “Grid Expression” and How to Combine Dynamics With Other Basic Training Angles

“Grid” as a graphical basis is the main limiting framework for dynamic expression in this practice. The main considerations are listed as follows: (1) Grid construction is an important form and content in graphic training. Grids not only have the characteristics of neat beauty, are simple and easy to accept but also have rich variability and diverse features when carrying out bone changes. Compared with free composition, grid composition not only appears to be traceable but also has enough space to play (Djonov and Van Leeuwen, 2013; Lee et al., 2014). (2) Dynamic expression is an extremely broad content. In the process of combining training with abstract graphics, the existence of a grid provides a basis for a dynamic framework. For students who are more confused about dynamics in the early stage, they can form a “ladder of understanding and application,” and the basic unit of the grid, the “cell shape,” can help students understand the “composite dynamic” organizational relationship between “elements” that comprise “units” and “units” that comprise “arrays” (Bokil and Ranade, 2014). (3) Based on the grid, the “morphological layer” and “organization layer” are also rich in expressiveness, providing a wealth of expandable space for advanced expression.

Of course, just as “skeletal composition” is only one method of graphic composition, the dynamics of the grid are only a form of dynamic innovative expression. Basic training can assist in the establishment of basic thinking about dynamic expression to a certain extent. However, dynamic innovative expression also has more dimensions and can be explored and practiced. In future research, dynamic expression training can also be carried out from more basic training perspectives.

### As the Subject of Training, Whether Emotional Performance Has Value and Significance

In this practice, in addition to the exploration and practice of “dynamic expression” from thinking to structure to mode, a small proposition of “how dynamic expression conveys emotion” was proposed at the back end of the training and put into practice. Dynamic training is an exploration of means. For example, in the course, a homemade program by Unity3D helps students clearly distinguish the morphological layer, motion layer and organization layer in the course. Through group simulation animation and other methods to interpret a variety of skeletal decomposition methods that constitute the work and comb the movement order, all of them are ways to explore how to strengthen specific methods of dynamic expression. These methodological explorations can help students learn to observe dynamics and understand the composition of a rich dynamic, thereby gradually establishing a “dynamic” thinking mode. However, as the theme of training, emotional expression is considered from the perspective of connotation. Although how to realize and organize dynamics is not addressed, how to form multiple possibilities in the way of “motion” is discussed. This approach puts a higher demand on dynamic organization skills, which we consider an indispensable part of basic training. Because the ultimate purpose of dynamic expression is “expression,” innovative expression must have attitude and emotional expression to arouse people’s interest and form public resonance and even have a certain value of artistic expression (Keltner et al., 2019). The training limited to means may be mechanical or feeble, although the emotional expression presented in the final work is not very precise and even immature. However, in the process of exploration, the consideration of comprehensive factors, such as how to grasp the precise acceleration, the combined motion of form, and the relationship between color changes in the motion can help students establish a dynamic innovative expression driven by emotional expression and cultivate students’ dynamic expression of innovative ideas and entrepreneurial thinking (Kamachi et al., 2013). This approach promotes the further combination of technical means and artistic expression.

## Conclusion

From the analysis of the course process and results, it can be seen that the digital media environment has unknowingly affected the way that the public receives information. As a major of digital media art under design science, the largest difference between it and the traditional design major lies in the rich sensory channels of the design object that can be employed as a multilanguage system for design display and artistic expression. In the results of questionnaire survey analysis, some results often exceed expectations, which mainly revolve around the contrast between recognition in design consciousness and uncertainty in clear goals. In the consciously guided experimental group, the clear goal and implementation awareness was stronger than in the control group, but in the pure vague consciousness group, which could be nurtured by daily experience, the control group even exceeded the experimental group. This part of the difference was not anticipated before the start of the experiment, but it truly reflects the impact of the current digital age on the visual literacy of the general public, especially the sensitive youth groups that have grown up in this era.

However, the cultivation of artistic design talent cannot only be based on ignorant consciousness. For professionals, it is necessary to transform vague consciousness into expressional measures that have professional expression and vary based on satisfactory and professional media literacy. Therefore, it is necessary for basic courses, especially those on modeling ability, to explore more abundant and practical models based on the characteristics and needs of the digital media faced by this future professional group. Dynamic innovative expression arises with the demand of digitalization, intelligence of media, and complex and high efficiency of information transmission (Park and Nam, 2007). Dynamic innovative expression also has a profound impact on future professionals who are immersed in digital media; it is not only the natural language environment of this group but also an important means of future artistic expression. Therefore, the cultivation of dynamic innovative expression design ability should be transformed from specific professional direction or ability direction training to a cultivation of the basic ability facing all-oriented design professionals, especially digital media art design professionals. In this way, the cultivation of the lowest level and the establishment of consciousness can maintain the consciousness of dynamic expression and establish a dynamic expression language system in a more subdivided professional field, establish the cognitive literacy of pan-digital media by using innovative concepts and entrepreneurial education, and even form a unique and stylized dynamic language.

## Data Availability Statement

The original contributions presented in the study are included in the article/supplementary material, further inquiries can be directed to the corresponding author/s.

## Ethics Statement

The studies involving human participants were reviewed and approved by the Jiangnan University. Written informed consent for participation was not required for this study in accordance with the national legislation and the institutional requirements.

## Author Contributions

JZ, MZ, YL, RL, and RC developed the theoretical framework and model in this work and drafted the manuscript. All authors contributed to the article and approved the submitted version.

## Conflict of Interest

The authors declare that the research was conducted in the absence of any commercial or financial relationships that could be construed as a potential conflict of interest.

## Publisher’s Note

All claims expressed in this article are solely those of the authors and do not necessarily represent those of their affiliated organizations, or those of the publisher, the editors and the reviewers. Any product that may be evaluated in this article, or claim that may be made by its manufacturer, is not guaranteed or endorsed by the publisher.
